# Maximizing diversity within and among teams in a large-scale project

**DOI:** 10.1016/j.mex.2022.101908

**Published:** 2022-11-04

**Authors:** Irit Talmor

**Affiliations:** The Sir Harry Solomon School of Management, Western Galilee College, Akko, Israel

**Keywords:** Assignment problems, Integer linear programming, Penalty function, Binary search

## Abstract

This work aims to improve an earlier methodology for assigning personnel to diverse three-member teams. Notably, the original algorithm focused only on diversity within teams, to ensure that conflicting interests are represented in each team. While this indeed created diverse teams, in many cases different teams featured the same combination of conflicting interests. The client for the original project, a government agency, asked for a methodology that produces more combinations. Hence, the current study presents an approach for boosting diversity among teams. That is, the new method maximizes the differences among teams, not only differences within teams. This was achieved by limiting the number of times each combination appears, while maintaining maximal diversity within teams as well. The upgraded algorithm is scalable and fast converging.

• We suggest an integer linear programming algorithm for creating thousands of diverse three-member teams that represent the conflicting interests of different groups.
• The algorithm imposes penalty costs on potential assignments based on their deviations from the project's requirements and sets upper bound constraints on the frequency of different assignments.
• The algorithm is efficient, scalable, and converges to maximal diversity within seconds.

## Graphical abstract

**Figure:**
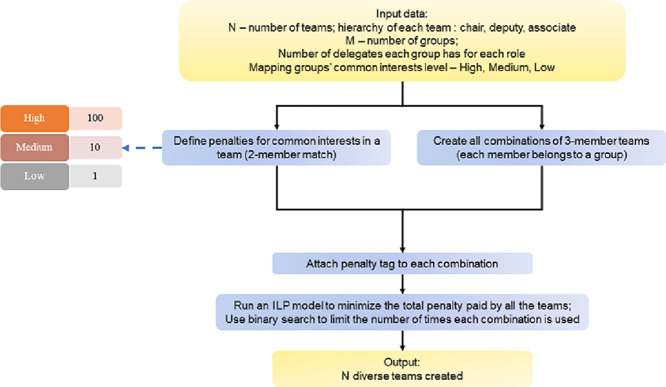

Specifications table

**Table:** 

Subject area:	Economics and Finance
More specific subject area:	Operations Research
Name of your method:	Assignment problems Integer Linear Programming
Name and reference of original method:	Talmor I. Solving the problem of maximizing diversity in public sector teams. *Socio Econ Plann Sci* 2022;81:101191. https://doi.org/10.1016/j.seps.2021.101191.
Resource availability:	N/A

## Introduction

Diversity plays an important role in many aspects of modern life – politics, business, education, etc. [1,2]. There are cases where such diversity is essential. For example, in politics, when different causes and interests compete for limited resources, or when mutual monitoring is required to prevent corrupt deals behind closed doors. Diversity is also necessary when staffing positions in committees and boards of public sector organizations.

The maximally diverse grouping problem (MDGP) is well-known in the literature. MDGP aims to assign a given set of elements (i.e., employees) to a fixed number of mutually disjoint subsets (i.e., teams) to maximize overall diversity (i.e., among members of the same team). The approaches for solving this problem include cross entropy [3], variable neighborhood search [4], iterated maxima search heuristic algorithm [5], a three-phase search [6], an iterated tabu search algorithm [7], a hybrid genetic algorithm [8] and many more. A variation of MDGP refers to cases where diverse teams need to be created from several homogenous groups. This sub-problem is often formulated as a multi-sided game [9], [10], [11] or as a k-partite graph [12,13]. This variation of MDGP has numerous applications in the real world. Thus, many case-specific algorithms and heuristics were developed for it. Examples include staffing hospital shifts [14], [15], [16], assigning flights to airport gates [17,18], matching teachers to schools [19,20], integrating experts from several fields in teams to increase creativity and innovation within organizations [21]; creating heterogeneous teams of students [22,23]; nominating students for exchange programs; and even assigning multiple targets for a swarm of unmanned aerial vehicles [24].

Besides Yeoh and Mohamad [23], whose approach is based on randomization, all the other abovementioned studies suggest binary variables models (i.e., each pair - element, team - is represented by a variable with a value of 1 if the element participates in the team and 0 otherwise). Then, heuristics algorithms are developed because these models are characterized by a rapid increase in run time. Both approaches – randomization and heuristics algorithms - do not guarantee optimal outcomes. Moreover, under the randomization approach, the gap between the optimal solution and the converged result is not necessarily bounded.

In a previous study, we suggested a different approach to maximizing the diversity of teams created from homogenous groups [25]. The novelty of our updated model lies in integrating two concepts: recognizing and prioritizing two forms of diversity, both within and among teams, and focusing on groups rather than on the individuals within groups. The first concept is integrated by defining diversity rules and setting a price tag for each rule so that its value is proportional to its priority. Thus, the requirement for maximal diversity is equivalent to the objective of minimizing the penalties. The second concept enables us to efficiently solve very large problems (i.e., creating a massive number of diverse teams). The current study improves the original algorithm by adding an iterative process that enables us to maximize diversity among teams as well. Both versions of the algorithm are efficient, scalable, and converge within seconds.

In Section 2, we present the problem that was the trigger for developing the algorithm. In Section 3, we present the original algorithm and apply it to an illustrative example. In Sections 4 and 5 we present the updated algorithm, apply it to the illustrative example and two case studies, and compare the solutions to demonstrate the improvement.

## Framing the Problem

The challenge we faced was to create thousands of diverse teams for a special project. Each team included three members: chair, deputy, and associate, selected from various interest groups. The number of groups could potentially range from 10 to 20, their size was proportional to their political power, and the total number of members in all groups was sufficient to fully staff all the required teams. There were three levels of shared interests among groups: high, medium, and low. High refers to groups whose interests match strongly; Medium applies to groups that share similar but not the same interests; Low applies to groups that share very limited or no interests. Members of the same group would naturally be designated as High.

The project's managers preferred that team members share as few common interests as possible to facilitate effective monitoring and increase the odds of optimal performance, which depended on minimizing improper cooperation between team members. In the past, creating such groups was based on a lottery and the greedy approach – that is, in each round three delegates were randomly selected. If this trio shared a low level of interests, the team was approved, the size of their groups was reduced by 1, and the next lottery was executed. Otherwise, the groups' sizes remained the same and another lottery trial was executed. This process was exhausting, time-consuming, and did not guarantee maximal diversity. Furthermore, there was no diversity benchmark for evaluating the results.

## The original algorithm

The basic algorithm we suggested to resolve the problem included three stages. First, all possible combinations for setting up three-member teams are listed. Second, a penalty function is calculated for each combination. Third, the mix of combinations that minimizes the total penalty is determined using integer linear programming (ILP).

### Stage 1 – Creating the list of combinations

The list of combinations is created using the existing groups: delegates from the first, second, and third groups are chosen for the roles of chair, deputy, and associate roles, respectively. At this stage, we do not restrict the team's makeup (i.e., several delegates from the same group may be chosen). Thus, given that there are *M* groups, the number of possible combinations is *M*^3^.

### Stage 2 – Defining the penalty function

First, based on management's preferences, we set price tags as follows: a pair of delegates from groups that share a high level of interests costs 111 points; a pair of delegates from groups that share a medium level of interests costs 11 points; a pair of delegates from groups that share a low level of interests costs 1 point. These price tags were chosen to reflect the fact that each level of interests includes the levels below it as well, and is thus penalized for it.

Second, we implemented this rule to define the price tag for each pair of groups according to their level of shared interests.

Third, we calculated the penalty incurred by each combination created in the first stage as the sum of the price tags of its pairs.

For example, suppose that there are four groups: A, B, C, D. Thus, there are 64 (=4^3^) combinations in the list – starting with (A, A, A) and ending with (D, D, D). Also, assume that the map of interests is as follows: Only pairs of delegates from the first group – such as (A, A), (B, B), etc. – share a high level of interests, so their price tag is 111; groups A and B share a medium level of interests, so the price tag of (A, B) and (B, A) is 11; groups C and D share a low level of interests, and therefore the price tag of (C, D) and (D, C) is 1.

Referring to the rules defined above, the penalties assigned to combinations in the list created in the first stage ranged from 333, for teams where all delegates belong to the same group, to 1, for teams comprising delegates from groups (A, C, D) or from groups (B, C, D). The complete penalty function and its frequency (i.e., the number of combinations assigned each penalty) are shown in Table 1.

**Table 1:** Penalty function (values and frequencies) for 3-member teams arranged from 4 different-sized groups: A, B, C, D, in line with a given map of interests: High – {A}, {B}, {C}, {D}; Medium – {A, B}; Low – {C, D}. The “price tag” of a team is based on the level of common interests among its members: high penalty for high level; medium penalty for medium level; and low penalty for low level.

Combination $\left(m_{1},\;\;m_{2},\;m_{3}\right)$	Demonstrations	Penalty	Frequency
$m_{1}=m_{2}=m_{3}$	(A, A, A), (B, B, B)…	333	4
$\left\{m_{1}\right\}\cup \left\{m_{2}\right\}\cup \left\{m_{3}\right\}=\left\{A,\;B\right\}$	(A, A, B), (A, B, B)…	133	6
$\left\{m_{1}\right\}\cup \left\{m_{2}\right\}\cup \left\{m_{3}\right\}=\left\{C,\;D\right\}$	(C, C, D), (D, C, D)…	113	6
$\left\{m_{1}\right\}\cup \left\{m_{2}\right\}\cup \left\{m_{3}\right\}=\left\{A,\;C\right\}$ or	(A, A, C), (C, C, A)…	111	24
$\left\{m_{1}\right\}\cup \left\{m_{2}\right\}\cup \left\{m_{3}\right\}=\left\{A,\;D\right\}$ or	(A, A, D), (D, D, A)…		
$\left\{m_{1}\right\}\cup \left\{m_{2}\right\}\cup \left\{m_{3}\right\}=\left\{B,\;C\right\}$ or	(B, B, C), (C, C, B)…		
$\left\{m_{1}\right\}\cup \left\{m_{2}\right\}\cup \left\{m_{3}\right\}=\left\{B,\;D\right\}$	(B, B, D), (D, B, D)…		
$\left\{m_{1}\right\}\cup \left\{m_{2}\right\}\cup \left\{m_{3}\right\}=\left\{A,\;B,C\right\}$ or	(A, B, C), (B, A, C)…	11	12
$\left\{m_{1}\right\}\cup \left\{m_{2}\right\}\cup \left\{m_{3}\right\}=\left\{A,B,\;D\right\}$	(A, B, D), (D, A, B)…		
$\left\{m_{1}\right\}\cup \left\{m_{2}\right\}\cup \left\{m_{3}\right\}=\left\{A,\;C,D\right\}$ or	(A, C, D), (D, A, C)…	1	12
$\left\{m_{1}\right\}\cup \left\{m_{2}\right\}\cup \left\{m_{3}\right\}=\left\{B,\;C,D\right\}$	(B, C, D), (D, C, B)…		

### Stage 3 - Integer linear programming

Next, integer linear programming is used to find the best mix of combinations, which maximizes the diversity of each team. This goal is equivalent to the objective of minimizing the total penalty imposed on all teams.

Because the penalties are derived from the teams' combinations, we defined the decision variables as follows: each decision variable represents the number of times a specific combination is assigned. This approach enables us to substantially reduce the size of the problem because the focus is not on the number of matches required, which may be tens of thousands, but on the number of possible combinations (which is the number of groups to the power of 3 – hundreds or a few thousands at most).

The problem's constraints include sets of equations – a set of three equations for each group. Each equation represents a role. The sum of decision variables that represent combinations in which this group holds this role is equal to its number of delegates for this role. The total number of constraints is three times the number of groups.

Referring to the illustrative example (section 3.2), assume that 1,000 teams should be assigned, and that the number of delegates for each role and group are as given in Table 2. The ILP formulation is as follows in section 3.4.

**Table 2:** Data of illustrative example - 4 different-sized groups A, B, C, D, and a given map of interests. Each group has delegates for each role. The mission is to create 1,000 3-member teams in a way that maximizes diversity in each team, while also maximizing diversity among teams.

	groups	A	B	C	D	Total
**Number of delegates**	**Chairs**	400	300	200	100	1000
	**Deputies**	300	400	100	200	1000
	**Associates**	250	250	300	200	1000
**Map of interests**	High {A}, {B}, {C}, {D} Medium {A, B} Low {C, D}					

### The ILP formulation of the illustrative example

**Given parameters**

*N* = 1,000 Number of teams
M={A,B,C,D} Set of groups
$B_{role}$ the number of delegates that each group allocates for each role:
$B_{chair}=\left(400,\;300,\;200,\;100\right)$
$B_{deputy}\;=\;\left(300,\;400,\;100,\;200\right)$
$B_{associate}=\left(250,\;250,\;300,\;200\right)$

For each vector, the sum of its components is equal to the number of teams:

$\sum_{m\in \left\{A,\;B,\;C,\;D\right\}\;}b_{m,role}=1000\;\forall \;role=chair,\;deputy,\;associate$
L_High={{A},{B},{C},{D}}
L_medium={{A,B}}
L_Low={{C,D}}

**Analyzed parameters**

*S *A vector that lists the 64 three-group combinations:
$S=\left(s_{1},s_{2},\ldots s_{64}\right)=\left(\left(A,\;A,\;A\right),\;\left(A,\;A,\;B\right),\;\ldots ,\;\left(D,\;D,\;C\right),\;\left(D,\;D,\;D\right)\right)$
$s_{j}=\left(s_{chair}\left(j\right),\;s_{deputy}\left(j\right),\;s_{associate}\left(j\right)\right)$
C The penalty costs of vector S, $c_{j}\;$is the cost for assigning one team by the $s_{j}$ combination, following the penalty function as shown in Table 1.
C=(333,133,…,113,333)

**Decision variables**

*X *A vector in which each element represents the number of teams in the j*-th* element of S; i.e., $x_{1}$ represents the number of teams where the combination is (A,A,A);
$x_{2}$ represents the number of teams where the combination is (A,A,B);
…
$x_{64}$ represents the number of teams where the combination is (D,D,D).
$X=\left(\begin{array}{c} x_{1}\\ x_{2}\\ \dot{\vdots }\\ x_{64} \end{array}\right)$

**Objective function**

(1) $$Z=Min\left\{333x_{1}+133x_{2}+\ldots +113x_{63}+333x_{64}\right\}$$

**Constraints**

Constraints for chair assignments:

(2) $$\sum_{\left\{s_{j\;}|s_{chair}\left(j\right)\;=A\right\}}x_{j}=400$$

(3) $$\sum_{\left\{s_{j\;}|s_{chair}\left(j\right)=B\right\}}x_{j}=300$$

(4) $$\sum_{\left\{s_{j\;}|s_{chair}\left(j\right)=C\right\}}x_{j}=200$$

(5) $$\sum_{\left\{s_{j\;}|s_{chair}\left(j\right)=D\right\}}x_{j}=100$$

Constraints for deputy assignments:

(6) $$\sum_{\left\{s_{j\;}|s_{deputy}\left(j\right)=A\right\}}x_{j}=300$$

(7) $$\sum_{\left\{s_{j\;}|s_{deputy}\left(j\right)=B\right\}}x_{j}=400$$

(8) $$\sum_{\left\{s_{j\;}|s_{deputy}\left(j\right)=C\right\}}x_{j}=100$$

(9) $$\sum_{\left\{s_{j\;}|s_{deputy}\left(j\right)=D\right\}}x_{j}=200$$

Constraints for associate assignments:

(10) $$\sum_{\left\{s_{j\;}|s_{associate}\left(j\right)=A\right\}}x_{j}=250$$

(11) $$\sum_{\left\{s_{j\;}|s_{associate}\left(j\right)=B\right\}}x_{j}=250$$

(12) $$\sum_{\left\{s_{j\;}|s_{associate}\left(j\right)=C\right\}}x_{j}=300$$

(13) $$\sum_{\left\{s_{j\;}|s_{associate}\left(j\right)=D\right\}}x_{j}=200$$

Value range:

(14) $$x_{j}\in N⋃\left\{0\right\},\;\forall j=1,2,\ldots ,64$$

The general mathematical formulation is given in the appendix.

## Results

The algorithm was coded in Python 3.7.2 on a conventional laptop. We applied it to the illustrative example and got the optimal solution of 10,000 penalty points within 0.68 seconds, distributed as follows: 900 teams with a price tag of 11, and 100 teams with a price tag of 1.

When zooming into the overall mix of teams, we noticed that only 9 combinations were used out of the possible 64. Furthermore, there was no balance among these 9 combinations – one combination was assigned 300 times whereas others were used only 50 times (see Table 3). Such outcome is justified when that is the only mix that minimizes the total penalty, but was it really the case? It is more likely that the model terminates when the minimum total penalty is achieved, because no limit was imposed on the frequency of combinations. This means that only one aspect of diversity (that is, within teams) is addressed in the original algorithm, and not a second aspect (diversity among teams). This drawback is discussed and solved in Section 4.

**Table 3:** Mix of combinations in the optimal solution produced by the original algorithm – illustrative example (maximizing diversity within teams).

Serial number in combinations vector	Combination	penalty	Number of times used (=number of teams created)
7	(A, B, C)	11	300
10	(A, C, B)	11	50
12	(A, C, D)	1	50
20	(B, A, D)	11	100
29	(B, D, A)	11	200
34	(C, A, B)	11	100
37	(C, B, A)	11	50
40	(C, B, D)	1	50
50	(D, A, B)	11	100
Total	10,000	1000	

## The Updated Algorithm

As noted, the concept of diversity can be applied within teams (assigning diverse delegates to a given team), but also among teams (producing teams featuring diverse combinations of delegates).

Although the original algorithm is efficient, scalable, and converges quickly, it only addresses the first concept of diversity mentioned above. As a result, a specific match with a minimal penalty may be used to produce many teams, while other combinations with minimal penalty scores may be used only a few times or not at all, even though the optimal values of the objective function in both cases are the same. In the long run, interest groups may exploit this drawback to promote improper cooperation. To prevent or limit this possibility, we suggest a solution that limits the number of teams featuring the same combination of delegates in a way that ensures diversity both within and among teams. This goal is achieved by adding an iterative process to stage 3 based on a binary search, as follows:

**Table:** 

Initialize:	a. Perform stage 1, stage 2, and stage 3 of the basic algorithm to determine Z and X. Z is the optimal value of the objective function, and X is the vector of instances (i.e., the number of times each combination is used).	
	b. Set:	
	$Q=\max_{j}\left\{x_{j}|x_{j}\in X\right\}$	Q is the largest element in X, i.e., the number of times the most popular combination is used.
	$L=\lceil \frac{N}{{\left|M\right|}^{3}}\rceil$	L is the minimal positive element in X. To guarantee a solution, the minimal number of times each combination is used should be at least the number of required teams divided by the number of potential combinations.
Step 1	Determine:	
	$R=|\left\{x_{j}|x_{j}\in X,\;\;x_{j}>0\right\}|$	R is the number of different matches in Xthat are used at least once.
Step 2	If |Q−L|≤0.01N	Terminate. The optimal solution is X, the number of combinations used is R, and the value of the objective function is Z.
Step 3	Calculate an upper bound: $UB=\frac{Q+L}{2}$	
Step 4	Add a set of inequalities to the original ILP model to create an updated model (hereafter – U-ILP): $x_{j}\leq Q1\;\forall x_{j}\in X$	
Step 5	Run the U-ILP model and get new Z1 and X1 (Z1 is the optimal value of the U-ILP objective function, and X1 is the vector of instances).	
Step 6	If Z1=Z set X≔X1, Q≔UB Else set L≔Q1,	Go to step 1 Go to step 2

The performance of the upgraded algorithm follows the performance of binary search $\log_{2}n$, where in our context n is the value of (Q−L), calculated in step 1b.

Applying the updated algorithm to the illustrative example increased the number of used combinations to 14 and balanced their frequency, as presented in Table 4. Whereas the difference between the most used and the least used combinations was 250 (300-50) in the original algorithm, it was only 88 (112-24) in the updated algorithm.

**Table 4:** Mix of combinations in the optimal solution produced by the updated algorithm – illustrative example (Maximizing diversity within AND among teams).

Serial number in combinations vector	Combination (chair, vice, associate)	penalty	Number of times used	
			Updated algorithm	Original algorithm
7	(A, B, C)	11	112	300
8	(A, B, D)	11	100	0
10	(A, C, B)	11	100	50
12	(A, C, D)	1	0	50
14	(A, D, B)	11	38	0
15	(A, D, C)	1	50	0
19	(B, A, C)	11	112	0
20	(B, A, D)	11	76	100
29	(B, D, A)	11	112	200
34	(C, A, B)	11	64	100
37	(C, B, A)	11	112	50
40	(C, B, D)	1	24	50
50	(D, A, B)	11	48	100
53	(D, B, A)	11	26	0
55	(D, B, C)	1	26	0
Total		10,000	1000	1000

## Implementation

The updated algorithm was applied to the two cases presented in Talmor [25]. The first case refers to creating 10,250 teams using delegates from 12 groups. The second case refers to creating 10,600 teams using delegates from 11 groups. The entire input data of these two cases is presented in Table 5.

**Table 5:** Input data of the two cases as presented in [25].

**Case 1**	**Groups**	A1	A2	A3	D1	D2	D3	G1	G2	H1	H2	H3	H4
**Number of delegates**	**Chairs**	2705	2896	874	662	500	443	396	335	314	255	237	633
	**Deputies**	2703	2899	866	668	499	438	401	342	311	249	234	640
	**Associates**	2704	2899	872	669	497	437	405	341	311	248	237	630
Map of interests*	**medium**	{A1, A2, A3}, {D1, D2, D3}, {G1, G2}, {H1, H2, H3, H4}											
	**Low**	{A1, A2, D1, D2, G1, H1, H2}, {A3, D3, G2, H3, H4}											

**Case 2**	**Groups**	A	B	C	D	E	F	G	H	I	J	K	
**Number of delegates**	**Chairs**	3276	531	622	798	621	182	267	264	1329	1151	1559	
	**Deputies**	3280	528	625	800	617	179	269	264	1331	1152	1555	
	**Associates**	3277	530	620	801	619	181	270	266	1330	1153	1553	
Map of interests*	**medium**	{A, B}, {C, D}, {E, F}, {G, H}, {J, K, F}											
	**Low**	{A, B, C, D, G, J}, {E, F, H, K}											

⁎ A high level of shared interests always exists with delegates from the same group. Also, the price tag of each pair is set according to its own level of shared interests as well as all levels below it.

Table 6 and Table 7 detail the process of the updated algorithm in cases 1 and 2, respectively.

**Table 6:** The iterative process of case study 1. Applying the updated algorithm to calculate maximal diversity within and among teams.

Iteration	Input		Output			Terminate?
	UB	Z1	Q	L	R	
Initialize	N/A	107312	669	8	35	No
1	339	107312	339	8	46	No
2	174	107414	339	174	N/A	No
3	257	107312	257	174	57	No
4	216	107312	216	174	68	No
5	195	107312	195	174	75	No
**6**	**185**	**107312**	**185**	**174**	**75**	**Yes**

**Table 7:** The iterative process of case study 2. Applying the updated algorithm to calculate maximal diversity within and among teams.

Iteration	Input		Output			Terminate?
	UB	Z1	Q	L	R	
Initialize	N/A	18820	1000	6	31	No
1	503	18820	503	6	46	No
2	255	18820	255	6	63	No
3	130	19518	255	130	N/A	No
4	192	18820	192	130	73	No
**5**	**161**	**18820**	**161**	**130**	**80**	**No**
6	146	19106	161	146	N/A	No
7	153	18938	161	153	N/A	Yes

In case study 1, the maximal diversity among teams was achieved in the 6th iteration. Note that the number of different combinations increased from 35 to 75, while the total penalty remained minimal. The process ended after approximately 20 seconds.

In case study 2, the maximal diversity among teams was achieved in the 5th iteration, where the gap between L and Q was bridged. The increase in the number of different combinations from 31 to 80 did not affect the total penalty value. The process ended after approximately 25 seconds.

**Fig. 1:**
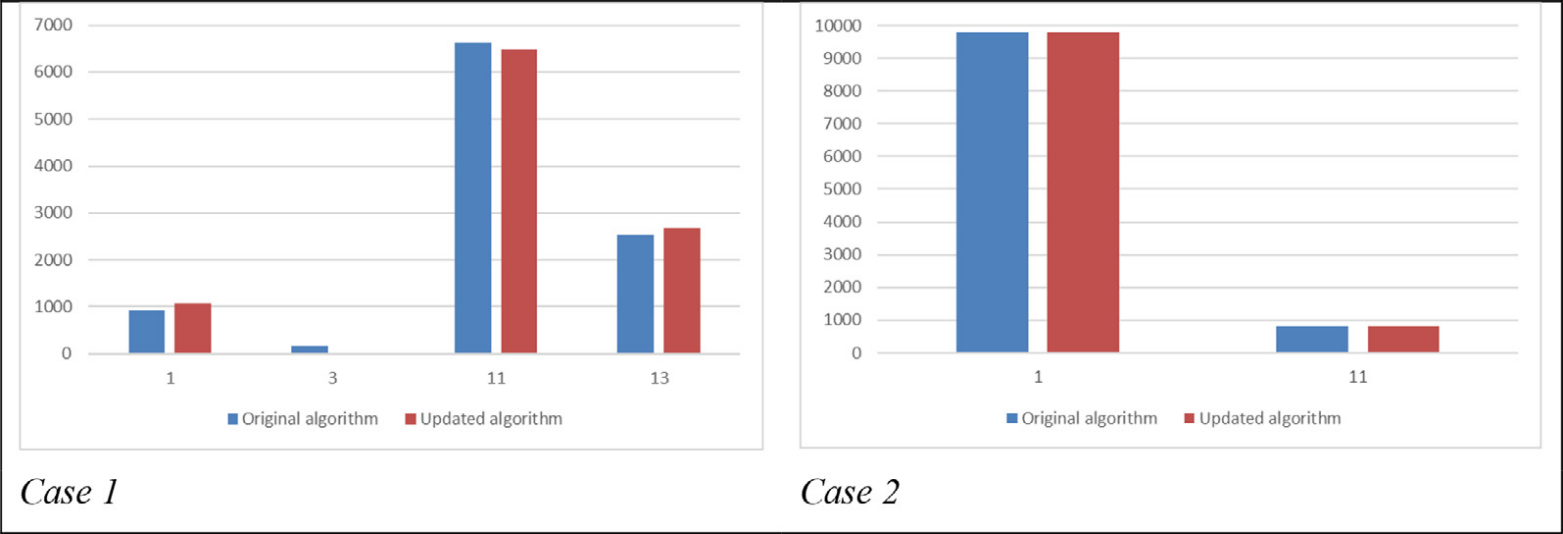
The price tag distributions – a comparison between the original and updated algorithms, cases 1 and 2.

When comparing the distributions of the price tags of the original algorithm solution vs. the updated algorithm solution, no substantial difference was observed. Fig. 1 demonstrates this finding.

## Discussion and Summary

This paper presents a practical and effective approach for creating thousands of diverse teams based on two notions: the first is the ranking of diversity preferences and setting penalties according to this rank; the second is referring to a group (and not to an element within it) as the basic entity when formulating the algorithm.

Thus, we set the stages of the algorithm as follows: first - identifying all possible team combinations; next - assigning penalty costs to each option, based on our diversity ranking; last - running an integer linear programming model formulated to minimize the total penalties and achieve an optimal set of assignments. An updated algorithm, based on the original one, was developed using an iterative binary search, so diversity was maximized both within and among teams.

Although the idea of a penalty may seem similar to previous studies that integrated multi-criteria analysis in the assignment process [20,26], the preferences for the ranking methods and mathematical formulation are different. Moreover, both the original and updated algorithms can be applied to problems involving very large numbers of assignments without increasing the convergence time to an optimal solution, as demonstrated in the case studies. The scalability and rapid convergence are rooted in the fact that the size of the problem depends only on the number of groups, rather than on the total number of groups members.

These benefits make our approach highly relevant in cases where heterogeneous teams are required (i.e., hospital shifts, educational activities, hackathons). Our model is also suitable for cases where team members must monitor each other and the number of teams with the same combination of members needs to be limited. Examples of such cases include counting election ballots or preventing security breaches at sensitive facilities.

As mentioned above, the number of groups impacts the scope of the problem: the larger this number, the more decision variables are required, increasing the run-time of the ILP model. In practice, the problems we coped with featured no more than 20 groups at most. Thus, the optimal solution was achieved in under 30 seconds for the updated algorithm. However, applying the algorithm to a problem involving a larger number of groups should be analyzed separately. More potential directions for future research include applying this approach to teams of different sizes or to larger teams.

## Declaration of Competing Interest

The authors declare that they have no known competing financial interests or personal relationships that could have appeared to influence the work reported in this paper.

## Figures and Tables

**Table 2:** 

**Parameters**		

N	Number of teams.	
M	Set of groups, namely: $M=\{m_{i}|\;m_{i}\;is\;the\;group^{\prime }s\;i\;name\}$	
$B_{\mathrm{role}}\;$	A vector that represents the number of eligible delegates of each group in a specific role. It is defined as: $B\left(\text{role}\right)=\left(b\left(m_{1},role\right),\;b\left(m_{2},role\right),\ldots ,\;b\left(m_{\left|M\right|},role\right)\right)\;\text{for}\;role=chair,\;deputy,\;associate$ Note that $B_{role}$ are non-negative integer vectors. In addition, for each vector, the sum of its components is equal to the number of teams: $\sum_{m_{i}\in M\;}b\left(m_{i},role\right)=N\;role=chair,\;deputy,\;associate$	
L_High	A set of sets of groups that share high level of interests, namely: $L\_High=\{L1_{k}|L1_{k}\subseteq M\}$	
L_Med	A set of sets of groups that share medium level of interests, namely: $L\_Med=\{L2_{k}|L2_{k}\subseteq M\}$	
L_Low	A set of sets of groups that share low level of interests, namely: $L\_Low=\{L3_{k}|L3_{k}\subseteq M\}$	
L_High,L_Med and L_Low are used to calculate the coefficient vector.		

**Analyzed parameters**		

S	A vector that lists all three-group combinations: $S=(s_{1},\;s_{2},\ldots ,s_{{\left|M\right|}^{3}})$ where: $s_{j}=\left(s_{chair}\left(j\right),s_{deputy}\left(j\right),s_{associate}\left(j\right)\right),\;$ for $s_{role}\left(j\right)\in M,\;role=chair,\;deputy,\;associate\;$	
C	The penalty costs of vector S, meaning that $c_{j}\;$is the cost for operating one team by the $s_{j}$ combination. $C=\left(c_{1},\;c_{2},\ldots ,c_{\left|S\right|}\right),\;c_{j}\geq 0\;$Following our penalty function as demonstrated in Table 3.	

**Decision variables**		

X	The vector of instances, i.e.: a vector in which each element represents the number of times the $s_{j}$ combination is used. $X={\left(x_{1},x_{2}\ldots ,x_{\left|S\right|}\right)}^{T}$	

**Objective function**		

$\;Z=Min\left\{\sum_{j=1}^{\left|S\right|}c_{j}x_{j}\right\}$		(A.1)

**Constraints**		

$\;\sum_{\left\{s_{j\;}|s_{role}\left(j\right)=m_{i}\right\}}x_{j}=b\left(m_{i},\;role\right)\;\text{for}\;role=chair,\;\text{deputy},\;associate,\;\forall m_{i}\in M$		(A.2)
$\;x_{j}\in N\cup \left\{0\right\},\;\forall j=1,2,\ldots ,\left|S\right|$		(A.3)

## Data Availability

Data will be made available on request.

## Appendix: Mathematical formulation

Eq. (A.1) is the mathematical formulation of the objective function; Eqs. (A.2) are the mathematical formulation of the constraints described in section 3.3: for each group $m_{i}$, the total number of teams in which this group holds a specific role equals to this group's allocation for this role. Eqs. (A.3) are the bounds of the combinations.
